# A dataset of aesthetic appeal ratings for pictograms with and without verbal labels

**DOI:** 10.3389/fpsyg.2026.1844763

**Published:** 2026-08-20

**Authors:** Naoto Ota

**Affiliations:** Department of Psychology, Aichi Shukutoku University, Nagakute, Aichi, Japan

**Keywords:** aesthetics, conceptual fluency, pictograms, processing fluency, symbol design, visual symbol

## 1 Introduction

In daily life, information is processed through language and through diverse visual symbols, including maps, public signage, and product icons. In non-English-speaking countries, such as Japan, their implementation has been actively promoted in public infrastructure, such as railway stations and airports (Kuroda, 2020). In Japan, a primary example of standardized visual symbols is the Design Principles of Pictorial Symbols for Communication Support (JIS T 0103) (hereinafter, JIS pictorial symbols) established by the Japanese Industrial Standards (JIS). Although compliance with JIS pictorial symbols is not mandatory, it ensures design consistency and mitigates the proliferation of disparate designs (Kuroda, 2020).

In contrast, creating “good pictograms,” rather than merely comprehensible ones, requires aesthetic quality (Kitagami, 2002). Modern design must balance aesthetic appeal with practical utility to ensure both effective communication and a positive user experience (Li, 2026), and the need to improve its aesthetic quality has been discussed in the public-health domain (Lujie et al., 2024). Indeed, when stimuli are abstract and unfamiliar, appealing icons yield better visual-search performance than unappealing ones (Reppa and McDougall, 2015), suggesting that aesthetic appeal may enhance information transmission efficiency in applied settings. Ota et al. (2025) assessed the comprehensibility of JIS pictorial symbols and found that most symbols satisfied the necessary condition of being easy to understand. However, no study has systematically assessed their aesthetic appeal.

Hence, this study developed normative data on the aesthetic appeal of JIS pictorial symbols, which would be a valuable resource for identifying the properties that determine the aesthetic appeal of visual symbols. Practically, visual symbols are presented either in isolation or in conjunction with verbal labels indicating their meaning. Therefore, this study assessed the aesthetic appeal via two surveys: one with labels and the other without. This would enable future researchers to select and apply the data according to the context of their work.

### 1.1 Processing fluency and aesthetic appeal

To examine the criterion-related validity of the survey data, this study investigated the relationship between the aesthetic appeal of visual symbols and semantic transparency (Kitagami et al., 2001; Kitagami and Muroi, 2006; Ota et al., 2025), an index of how easy visual symbols are to understand.

Although aesthetic evaluations of design are often assumed to rest on visual features such as simplicity, balance, and symmetry, these are also influenced by the semantic interpretation of the stimulus (e.g., Casteau and Smith, 2024). The processing fluency theory (Reber et al., 2004; Schwarz et al., 2021) postulates that the easier a stimulus is to process, the more positively it is evaluated (Graf et al., 2018; Winkielman et al., 2003; Reber et al., 2004; Yoo et al., 2024) because the positive metacognitive experience arising from fluent processing is misattributed to the stimulus (Jacoby et al., 1989; Lee et al., 2025; Yagi et al., 2023). Among the multiple components of fluency (Alter and Oppenheimer, 2009), this study focuses on conceptual fluency, that is, the ease of semantic processing, such as identifying a stimulus's meaning (Graf et al., 2018).

Symbols with high semantic transparency can be readily matched with the observer's conceptual knowledge, producing high conceptual fluency and, consequently, higher aesthetic evaluations (Hypothesis 1). Furthermore, this association is predicted to be stronger when the symbols are presented alone (Hypothesis 2). Explicit semantic information directs evaluators' attention to the conceptual aspects of the symbols, and the resulting reduction of uncertainty can enhance aesthetic appreciation (Duan et al., 2026). Conversely, without labels, evaluations and the effect of conceptual fluency should be relatively weaker. Confirmation of these directional predictions would support the criterion-related validity of the dataset.

## 2 Method

### 2.1 Participants

Participants were recruited via Yahoo! Crowdsourcing (https://crowdsourcing.yahoo.co.jp). They received compensation equivalent to ¥100. Both surveys were conducted in October 2025. Eligibility was restricted to native Japanese speakers aged ≥18 years. Participants' computer language settings were confirmed to be Japanese based on the metadata recorded during the survey. The sample size was determined to ensure that even with an anticipated 15% attrition rate from data cleaning, each item would receive 50–60 observations. Paisios et al. (2023) noted that several typical norming studies relied on approximately 30 observations per item, while arguing that such sample sizes may be insufficient to yield reliable distributions. Consequently, this study exceeded the typical observation count found in norming research.

Based on this rationale, approximately 200 participants were recruited for each survey. The study included 313 items, divided into three lists. Hence, recruiting approximately 200 participants per survey yielded approximately 66 observations per item (200/3 ≈ 66.7). Responses were obtained from 209 and 199 participants in the label-present and symbol-only surveys, respectively.

### 2.2 Stimuli

The stimuli consisted of 313 JIS pictorial symbols (JIS T 0103), each 450 × 450 pixels in size (72 DPI). In the label-present survey, symbols were accompanied by linguistic labels indicating their meanings. Both the images and linguistic labels were used exactly as provided in JIS T 0103 without modifications. The stimuli (JIS T 0103) are available at https://www.kyoyohin.org/ja/research/japan/jis_t0103.php.

One item from the Directed Questions Scale (DQS, Maniaci and Rogge, 2014) was embedded in each survey. In the DQS trials, an image of the same size as the symbol stimuli was presented instead of the symbol image. The DQS instruction was presented as an image displaying the Japanese instruction “Please be sure to select ‘3'.” In each survey, the 313 stimuli were divided into three lists. Since one DQS item was added to each list, the number of items per list ranged from 105 to 106. The same set of items was used in both surveys.

### 2.3 Procedure

The institutional ethics committee approved this study (approval number 2025-06). Two independent online surveys, a label-present survey and a symbol-only survey, were programmed and conducted using Qualtrics. Within each survey, participants were randomly assigned to one of three lists. All the participants provided informed consent via a web browser before participating in the study. Each trial presented one symbol (450 × 450 pixels, 72 DPI) at the center of the screen. In the label-present survey, the verbal label appeared directly above the symbol in 20-point font; the symbol-only survey was identical except that no label was presented. The question “How aesthetically appealing is this icon?” (in Japanese) appeared directly below the symbol, followed by the 6-point rating scale. All trials were self-paced, with no time limit imposed on individual ratings. A schematic illustration of the trial layout for both surveys is available in the OSF repository (see trial_layout_section.html).

Japanese instructions for aesthetic appeal were adapted from Collaud et al. (2022). Participants rated each symbol on a 6-point scale (1 = least appealing, 6 = most appealing). Participants were instructed to assign higher values to symbols that they judged as attractive and lower values to those judged as unattractive. Crucially, they were instructed to focus strictly on visual attractiveness, regardless of the symbol's inherent meaning.

Participants were instructed to complete the survey within 30 min, with short breaks permitted. Afterward, they provided demographic data (gender, age, education), received a debriefing, and were compensated. Yahoo! Crowdsourcing functionality strictly prevented repeated participation, ensuring no participant overlap between the two surveys.

### 2.4 Analysis plan

To ensure data quality, the following exclusion criteria were applied sequentially. Participants with extremely long completion times and those whose times deviated by more than ±2 standard deviations (SDs) from the mean were excluded. Furthermore, participants who failed the DQS or provided identical responses across all items (straight-lining) were excluded. Finally, participants whose rating patterns deviated from the overall trend were excluded. Specifically, the correlation between each participant's ratings and the overall mean was calculated, and those with correlations below 0.20 or negative values were excluded. This criterion was used in the norming study by Pexman et al. (2019) to exclude participants whose rating patterns deviated from the overall trend, and was subsequently adopted by Ota et al. (2025) in collecting the semantic transparency norms for the same 313 JIS pictorial symbols examined here. Applying the same criterion ensures methodological continuity with Ota et al. (2025), whose semantic transparency norms serve as the criterion measure for validating the present dataset.

Split-half reliability was calculated following Paisios et al. (2023) to examine the internal consistency of items across participant subsamples. In addition, to evaluate the precision of each item-level mean, the standard error (SE) was computed for every item, following the precision indexing approach of Desrochers and Thompson (2009). Once reliability was confirmed, individual ratings were averaged to compute the item-level mean aesthetic appeal scores. All subsequent analyses were conducted at the item level. To test the hypotheses, correlations were calculated between aesthetic appeal ratings and the item-level semantic transparency values reported by Ota et al. (2025). Semantic transparency, measured on a 7-point scale (1–7), reflected symbol comprehensibility, with higher values indicating greater ease of understanding (Kitagami et al., 2001; Ota et al., 2025). To address Hypothesis 2, the correlation coefficients from the two surveys were compared using Steiger's (1980) method to determine whether the relationship was significantly stronger in the label-present survey than in the symbol-only survey.

## 3 Analysis

### 3.1 Data cleaning

Based on response time, three participants were excluded for extremely long completion times, exceeding 12 h, as they were judged unlikely to have engaged appropriately with the task. Subsequently, participants whose completion times deviated by more than ± 2 SDs from the mean within each survey were excluded as outliers. This resulted in the removal of eight participants from the label-present survey and six from the symbol-only survey. Moreover, participants who failed to select the specified numeric value “3” on the DQS were excluded. This excluded three participants from the label-present survey and two from the symbol-only survey. Subsequently, one participant who provided straight-lining responses in the symbol-only survey was excluded.

Each participant's rating pattern was evaluated by computing the correlation between their individual ratings and the item-level means of their assigned lists. Participants with negative correlations or below 0.20 were excluded. Based on this criterion, 25 participants were removed from the label-present survey and 21 from the symbol-only survey. Following all exclusions, data from 171 participants in the label-present survey and 168 participants from the symbol-only survey were included in the analysis. Participants' mean age was 50.43 years (SD = 11.69) for the label-present survey and 52.03 years (SD = 11.92) for the symbol-only survey. Regarding gender, the label-present survey included 126 men, 44 women, and 1 unreported, and the symbol-only survey included 122 men, 45 women, and 1 unreported. Detailed demographics, including age histograms and educational levels, are available in the OSF repository.

### 3.2 Split-half reliability

To evaluate internal consistency, split-half reliability was calculated for both surveys following Paisios et al. (2023). All stimuli, excluding the DQS items, were included in the analysis using 1,000 iterations per survey. In each iteration, participants were randomly divided into two groups per item to compute the group-specific mean ratings. The correlation between these group means was calculated and adjusted using the Spearman–Brown prophecy formula. After 1,000 repetitions, the mean and SD of the corrected coefficients were computed. In the label-present survey, the mean Spearman-Brown-corrected correlation was 0.925 (SD = 0.006). For the symbol-only survey, the mean was 0.911 (SD = 0.007). Both surveys yielded highly corrected coefficients, indicating stable internal consistency.

### 3.3 Stability of item-level means

Split-half reliability indexes the internal consistency of the ratings rather than the precision of individual item means. The standard error (SE) of each item mean was therefore computed. The median SE was 0.151 (label-present) and 0.143 (symbol-only) on the 6-point scale, equating to 3.0% and 2.9% of the scale range, respectively. After scaling to the rating range, these values are comparable to those reported by Desrochers and Thompson (2009; SE =0.14-0.18, 2.3-3.0% of the 7-point scale range). The comparable precision, despite a smaller per-item sample size in the present study (median *n* = 55-57 vs. *n* = 72), indicates that the item-level mean ratings are stable across independent participant subsamples. Per-item bootstrap 95% confidence intervals (5,000 resamples) are provided on OSF.

### 3.4 Distribution

**Figure 1:**
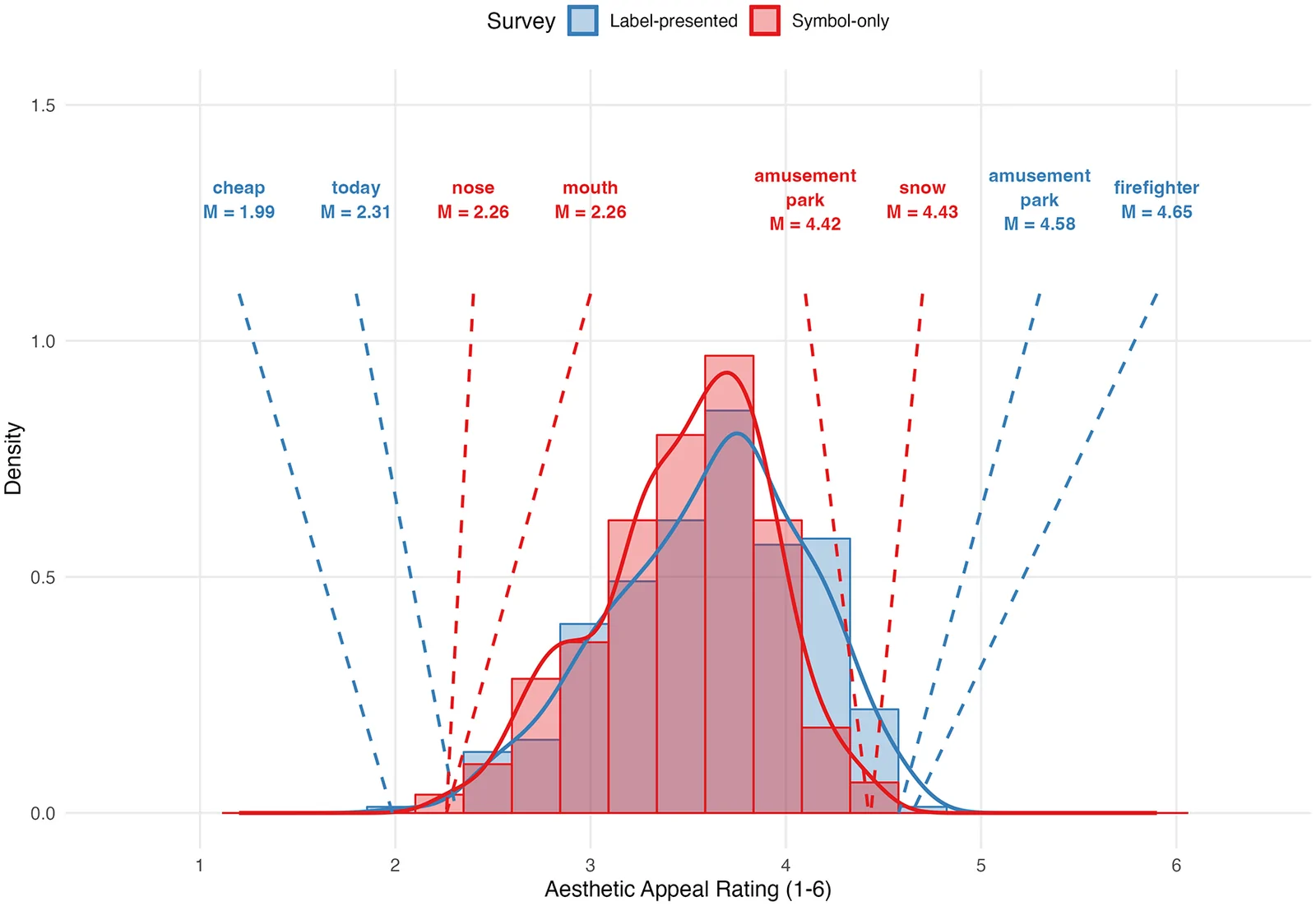
Histograms of aesthetic appeal ratings for the label-present and symbol-only surveys. The icon names and their corresponding mean ratings are shown as text.

The distribution of the datasets from both surveys is presented in Figure 1. Both followed a unimodal pattern, peaking around the scale's midpoint. However, in both surveys, the distributions were negatively skewed, with a long tail toward the lower end of the scale, indicating that participants generally assigned lower rather than higher attractiveness ratings to the symbols.

### 3.5 Correlations between the two aesthetic appeal ratings and semantic transparency

Correlation coefficients were computed to evaluate the relationship between semantic transparency and aesthetic appeal (Table 1). The results revealed a positive correlation in both surveys. To test whether the correlation between aesthetic appeal and semantic transparency differed between the two surveys, the coefficients were compared using Steiger (1980) method, with the *R* package cocor (Diedenhofen and Musch, 2015). The difference was significant (Δ*r* = 0.33, *z* = 11.64, *p* < 0.001), indicating that semantic transparency showed a strong positive correlation with aesthetic appeal in the label-present survey; however, a moderate correlation in the symbol-only survey.

**Table 1:** Pearson correlation coefficients between aesthetic appeal ratings (label-present and symbol-only) and semantic transparency.

	Labeled	Symbol only	Transparency
Labeled	–		
Symbol only	0.735^**^	–	
Transparency	0.802^**^	0.47^**^	–

Labeled indicates aesthetic appeal ratings from the label-present survey; Symbol only indicates ratings from the symbol-only survey; Transparency indicates semantic transparency from Ota et al. (2025). ^**^p < 0.01.

### 3.6 Sensitivity analysis

Re-running the analyses without the data-driven exclusion criteria (±2 SD completion time and correlation between individual ratings and the item-level means < 0.20; *N* = 203/195) yielded near-identical item-level means (*r* = 0.99 in both surveys), split-half reliability (0.92/0.90), Pearson correlations with semantic transparency (*r* = 0.81/0.47), and Steiger's test for the survey difference (Δ*r* = 0.33, *z* = 11.82, *p* < 0.001), indicating that the data-driven criteria did not meaningfully alter the normative values or the conclusions.

### 3.7 Effects of age and gender

Linear mixed-effects models were fitted to the trial-level ratings of each survey, with standardized age and gender (male vs. female) as fixed effects; the random-effects structure was selected by backward elimination from the maximal structure using the buildmer package (Voeten, 2026; final structures are reported in the OSF repository). Participants who did not report a binary gender (one per survey) were not included in these models because their numbers were too small to yield stable estimates. Age was not significant in either survey (label-present: *b* = −0.01, *p* = 0.840; symbol-only: *b* = −0.08, *p* = 0.112). Gender was also not significant in the label-present survey (*b* = −0.05, SE = 0.126, *p* = 0.716; male: *M* = 3.58, SD = 1.30; female: *M* = 3.63, SD = 1.16). In the symbol-only survey, however, male participants gave higher ratings than female participants (*b* = 0.23, SE = 0.115, *p* =0.047; male: *M* = 3.53, SD = 1.13; female: *M* = 3.34, SD = 1.27). Item-level means computed separately by gender are therefore shared in the Supplementary Material; because female participants were fewer, these values are less stable than the aggregate norms.

## 4 Dataset overview

This study collected aesthetic appeal ratings for 313 JIS pictorial symbols, a standardized set in Japan. Two surveys were conducted: a label-present survey, where symbols were accompanied by their meanings, and a symbol-only survey, where symbols were presented in isolation. The reliability analyses confirmed that both surveys yielded sufficiently high internal consistency. Consistent with Hypothesis 1, aesthetic appeal and semantic transparency were positively correlated in both surveys; the moderate correlation in the symbol-only survey suggests that participants inferred the underlying concepts even when symbols were presented in isolation (Graf et al., 2018). Consistent with Hypothesis 2, this correlation was significantly stronger in the label-present survey. The confirmation of these directional predictions provides evidence for the criterion-related validity of the dataset.

However, when utilizing this dataset, researchers should take into account the influence of participant gender, particularly for the ratings obtained in the symbol-only survey, in which male participants gave slightly higher ratings than female participants. Because the dataset includes participant-level, trial-level rating data, researchers can control for the effect of gender as needed, for example, by including it as a covariate or by computing subgroup-specific norms.

### 4.1 Limitations and future directions

The survey environment did not fully mirror real-world settings: whereas symbols are encountered in practice under varying visibility conditions and limited exposure durations, this study used high-visibility white backgrounds with unrestricted viewing time, potentially limiting ecological validity. For instance, while monotonous designs are considered less aesthetically appealing (Berlyne, 1971; Sun and Firestone, 2022), simple, high-visibility stimuli may be more resilient to aesthetic degradation under poor viewing conditions encountered in real-world settings. Future research should therefore examine aesthetic appeal in more diverse contexts.

Furthermore, because participants were adult native Japanese speakers recruited via a single crowdsourcing platform, the target population of these norms should be regarded as adult Japanese crowdsourcing users. Generalization to the broader Japanese population, or to other languages and cultures, requires further empirical evidence of representativeness. Future research should expand the sample to include diverse languages and cultural backgrounds, as well as populations with limited or no internet access.

Finally, future research should extend the present dataset by collecting ratings of visual complexity, concreteness, and emotional valence for the same set of 313 JIS pictorial symbols examined in this study. Although no such norms currently exist for the JIS pictorial symbols, these variables may influence aesthetic ratings (e.g., Collaud et al., 2022; McDougall et al., 2016); researchers should therefore consider their potential impact when utilizing this dataset. The primary aim of the present study was to develop and validate normative aesthetic appeal ratings, and identifying the variables that determine the aesthetic appeal reported in these norms remains an important task for future research.

In this study, a reliable and valid dataset of aesthetic appeal ratings was developed for JIS pictorial symbols, which represented one of the most prominent sets of visual symbols in Japan. As this dataset included ratings obtained both when symbols were presented with their meanings and when they were presented alone, it could be flexibly used in future research depending on the context of interest. Future studies may examine the relationships between aesthetic appeal and other theoretically relevant variables, which could clarify the factors that enhance or diminish the aesthetic appeal of symbols and provide empirical guidance for the design of new visual symbols.

## Data Availability

The datasets generated and analyzed for this study can be found in the Open Science Framework (OSF) repository at: https://osf.io/mb7qa/. Supplementary materials available in the repository include aggregated and trial-level datasets, analysis scripts, and experimental instructions. A README.html file is provided in the repository, which contains descriptions of all files and variables in the dataset.
